# Effects of Ultrasound-Guided Administration of Botulinum Toxin (IncobotulinumtoxinA) in Patients with Lateral Epicondylitis

**DOI:** 10.3390/toxins11010046

**Published:** 2019-01-15

**Authors:** Antonio Galván Ruiz, Gloria Vergara Díaz, Beatriz Rendón Fernández, Carmen Echevarría Ruiz De Vargas

**Affiliations:** 1 Physical Medicine and Rehabilitation, Hospital Universitario Virgen del Rocío, 41013 Seville, Spain; glo.vergara@gmail.com (G.V.D.); rendin@Hotmail.com (B.R.F.); carmenerv@telefonica.net (C.E.R.D.V.); 2 Pharmacology, Pediatrics and Radiology Department, University of Seville, 41004 Seville, Spain; 3 Motion Analysis Laboratory, Spaulding Rehabilitation Hospital, Department of Physical Medicine and Rehabilitation, Harvard Medical School, Boston, MA 02129, USA

**Keywords:** lateral epicondylitis, tennis elbow, botulinum toxin A, incobotulinumtoxinA, pain, rehabilitation

## Abstract

How effective and safe are incobotulinumtoxinA injections in adult patients with lateral epicondylitis refractory to other treatments? In this experimental study, ultrasound-guided incobotulinumtoxinA 10–30 U/muscle was injected into extensor carpi ulnaris, extensor digiti minimi, extensor digitorum longus and extensor carpi radialis brevis muscles. Pain (visual analogue scale [VAS], 0 to 10 [no pain to severe pain]) and upper-limb functionality (QuickDASH scale, 0 to 100 [best to worst]), assessed at baseline, 1, 3 and 6 months post-treatment, were analysed using repeated-measures analysis of variance (ANOVA) and Tukey post-hoc tests. Secondary analyses stratifying patient population by sex and baseline VAS were performed. Adverse events were reported. Twenty-four patients (mean [standard deviation] age 46.8 years) were included. Compared with baseline, mean VAS and QuickDASH scores improved at all follow-ups (*p* < 0.001 and *p* = 0.001, respectively; repeated-measures ANOVA). Secondary analyses revealed significant differences between baseline and all follow-ups in the group with baseline VAS ≥ 6 and in males and females (all *p* < 0.05, Tukey post-hoc test). No adverse events, except for the expected third finger weakness, were reported. In conclusion, ultrasound-guided incobotulinumtoxinA injections may be an effective treatment for lateral epicondylitis in the appropriate patient population.

## 1. Introduction

Lateral epicondylitis, commonly known as “tennis elbow,” is the main cause of elbow pain [1] and has been reported to affect 1% to 3% of adults each year [2,3]. The condition is initially caused by inflammation secondary either to a direct traumatic injury or to repeated functional overuse of the extensor carpi muscles, which are inserted into the lateral epicondyle of the humerus. The muscles involved are the extensor carpi ulnaris, the extensor digiti minimi, the extensor digitorum longus and the extensor carpi radialis brevis. Severe cases with repeated, strong overexertion may also affect the supinator muscle and elbow extensor muscles such as the anconaeus. The lesions may affect the insertional entheses, tendons or may even produce pain on the ventral side of the affected muscle, depending on the severity and stage of progression. Excessive traction on the muscle–tendon complex is a well-known pathophysiologic element for establishment of these lesions and progression to a chronic state [2,3,4]. For this reason, immobilizing splints and elbow straps have been used as conservative treatment in order to reduce traction on the injured tissues. 

BoNT-A, produced by *Clostridium botulinum*, is one of seven known BoNT serotypes that exerts its therapeutic effects by blocking the release of the neurotransmitter acetylcholine at cholinergic nerve endings, resulting in transient inhibition of involuntary muscle activity [5]. As such BoNT is an effective and recommended treatment for a range of medical conditions, including movement disorders (spasticity and dystonia) [6] and is also used to manage pain associated with excessive muscle contractions [5]. Reversible inhibition of muscle contraction by injection of a BoNT product may have a therapeutic role in the recovery of very long-standing lateral epicondylitis lesions in which, for many cases, surgical intervention is the only solution. Previously published evidence suggests that BoNT treatment is superior to placebo in lateral epicondylitis refractory to other treatments [4,7,8,9], with an average duration of effect of around 3 months and weakness of the third finger as the main undesirable effect [10]. However, clinical trials have shown variability in the duration of the effects, with clear recommendations to assess the specific muscles involved [11,12]. 

In this experimental study we used ultrasound guidance for the treatment of lateral epicondylitis, which increases the precision of treatment to symptomatic muscles, minimizing the risk of adverse events. Our objectives were to assess the efficacy of ultrasound-guided incobotulinumtoxinA (Xeomin^®^; Merz Pharmaceuticals GmbH, Frankfurt am Main, Germany) treatment in patients with lateral epicondylitis refractory to other conservative treatments, such as physiotherapy, electrotherapy, local injection of corticoids and proliferation therapy and to assess the safety profile in this group of patients. We hypothesized that BoNT would lead to a significant decrease in the severity of pain that would, in turn, facilitate the functional performance of the upper limb, thus leading to better clinical outcomes. In addition we sought to identify patient populations that would experience the greatest benefit from BoNT treatment.

## 2. Results

### 2.1. Demographics and Baseline Characteristics

Patient demographics and baseline characteristics are displayed in Table 1. Twenty-four patients met the study inclusion criteria. Most patients were female and the mean (standard deviation [SD]) age was 46.8 (9.0) years (Table 1). All patients showed dominance on their right side and the disease affected mainly the right side in most patients. All patients had received previous ineffective treatments including analgesics/non-steroidal anti-inflammatory drugs, physiotherapy, electrotherapy and peritendinous injections. The mean (SD) symptom progression time from initial onset was 20.0 (19.8) months (range 2 to 90 months). At baseline, 87.5% of patients presented with positive resisted manoeuvres in the extensor digitorum longus, 83.3% in the extensor carpi radialis brevis, 41.7% in the extensor digiti minimi and 29.2% in the extensor carpi ulnaris. A baseline mean (SD) pain intensity of 6.9 (1.8) was reported using a visual analogue scale (VAS) and the mean (SD) baseline score for upper limb functionality for all patients was 60.1 (20.9) using the QuickDASH scale. None of the patients experienced complete tendon rupture during this study.

### 2.2. Treatment

A total of 12 patients (50%) received incobotulinumtoxinA injections into more than 1 muscle. Doses administered per muscle were predefined based on muscle size (Table 2) and, although a maximum total dose of 80 U was permitted, patients were only treated in symptomatic muscles; therefore, no patient required the maximum dose.

### 2.3. Efficacy

**Figure 1:**
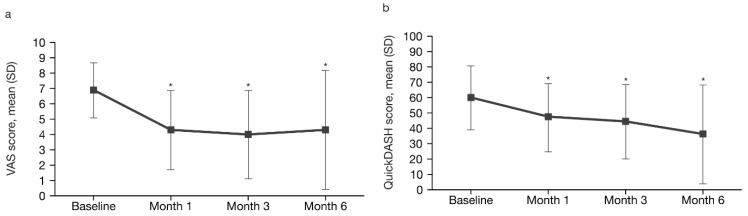
Pain intensity and functionality by visit: (**a**) VAS score (pain); and (**b**) QuickDASH score (functionality). * Statistically significant difference between baseline and follow-up assessments at Months 1, 3 and 6; Tukey post-hoc analysis; *p* < 0.05. VAS range: 0 (best) to 10 (worst); QuickDASH scale range: 0 (best) to 100 (worst). SD, standard deviation; VAS, visual analogue scale.

Pain intensity (VAS) decreased significantly from a mean (SD) of 6.9 (1.8) at baseline to 4.3 (2.6), 4.0 (2.9) and 4.3 (3.9) at the first, second and third follow-up assessments, respectively (*p* < 0.001; repeated-measures analysis of variance [ANOVA], Figure 1a). Post-hoc analysis indicated that VAS scores at all 3 post-treatment assessments were significantly different from baseline (*p* < 0.05).

Functionality (QuickDASH score) also improved significantly from a mean (SD) of 60.1 (20.9) at baseline to 47.6 (22.2), 44.5 (24.2) and 36.3 (32.3) at the first, second and third follow-up assessments, respectively (*p* = 0.001; repeated-measures ANOVA, Figure 1b). Post-hoc analysis indicated that QuickDASH scores at all 3 post-treatment assessments were significantly different to baseline (*p* < 0.05). 

Following the study: In 3 patients (12.5%), a repeat injection of incobotulinumtoxinA was required due to a positive but short duration, treatment effect. In a further 5 patients (20.8%), the effects of BoNT were not sufficient to recover normal functionality, thus the patients were required to continue on to surgical assessment. The data reported in this paper are only related to the first BoNT injection.

### 2.4. Analysis in Different Patient Groups

#### 2.4.1. Patients Grouped by Baseline VAS Score

We stratified the patient population according to baseline VAS score: (i) VAS score ≥ 6 (18/24 patients [75%]) and (ii) VAS score < 6 (6/24 patients [25%]). We performed a 2-way mixed ANOVA, in which the between-subject factor was chosen according to the baseline VAS score (2 levels) and the within-subject factor was the VAS score at all assessment visits (baseline and 3 follow-up assessments, i.e., 4 levels). Significant differences were seen between the 2 groups according to VAS scores at baseline and the remaining visits (*p* < 0.05). 

**Figure 2:**
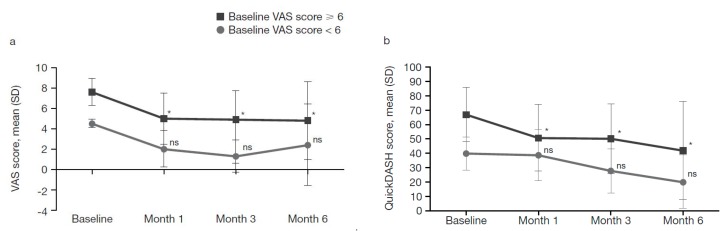
Pain intensity and functionality by visit and baseline VAS score: (**a**) VAS score (pain); and (**b**) QuickDASH score (functionality). * Statistically significant difference between baseline and follow-up assessments at Months 1, 3 and 6; Tukey post-hoc analysis; *p* < 0.05. Median VAS score at baseline and Months 1, 2 and 3, respectively: VAS score ≥ 6, 7.2, 5.0, 5.0, 4.8; VAS score < 6, 4.5, 2.0, 1.0, 0.5. Median QuickDASH score at baseline and Months 1, 2 and 3, respectively: VAS score ≥ 6, 72.5, 48.9, 52.5, 29.0; VAS score < 6, 45.0, 42.5, 23.5, 14.0. VAS range: 0 (best) to 10 (worst); QuickDASH scale range: 0 (best) to 100 (worst). ns, not significant; SD, standard deviation; VAS, visual analogue scale.

The post-hoc Tukey test also showed that within the group with baseline VAS scores ≥ 6, there was a significant difference between baseline and first, second and third follow-up assessments (*p* < 0.05 for all). However, there was no significant difference between the baseline VAS score and the scores at the first, second or third follow-up assessments in the group with VAS scores < 6 (*p* > 0.05 for all; Figure 2a).

Analysis of simple effects using the Tukey post-hoc test revealed significantly different VAS scores between the groups for baseline, first and second follow-up assessments (*p* < 0.05 for all) but not for the third (*p* > 0.05).

We also investigated the differences in QuickDASH scores between the 2 groups. A further 2-way mixed ANOVA, where the between-subject factor was the group according to the baseline VAS score (2 levels) and the within-subject factor was the QuickDASH score at all assessment visits (4 levels), showed main significant effects for both groups according to baseline VAS score and QuickDASH score (*p* < 0.05), indicating that both groups showed significant differences in QuickDASH scores across the 4 time points. The Tukey post-hoc test revealed significant differences between baseline and first follow-up, baseline and second follow-up and baseline and third follow-up assessments (*p* < 0.05 for all) in both groups. 

Analysis of the simple effects using the post-hoc Tukey test showed no significant differences in QuickDASH scores between the groups at baseline, first, second or third follow-up assessments (*p* > 0.05 for all). The post-hoc Tukey test also showed that within the group with baseline VAS scores ≥ 6, the QuickDASH scores were significantly different between baseline and first follow-up, baseline and second follow-up and baseline and third follow-up assessments (*p* < 0.05 for all). However, no significant differences between the baseline QuickDASH scores and the scores at follow-up assessments in the group with baseline VAS scores < 6 were shown (*p* > 0.05; Figure 2b).

#### 2.4.2. Patients Grouped by Sex

We stratified the population according to sex and performed a sub analysis of VAS scores using a 2-way mixed ANOVA in which the between-subject factor was sex (2 levels) and the within-subject factor was pain level (VAS scores) at the assessment visits (baseline and 3 follow-up assessments, i.e., 4 levels). Main significant effects were found for sex and VAS score (*p* < 0.05), indicating significant differences in VAS scores across the 4 time points in both groups. The post-hoc Tukey test revealed significant differences between baseline and first, second and third follow-up assessments (*p* < 0.05 for all) in both males and females. 

**Figure 3:**
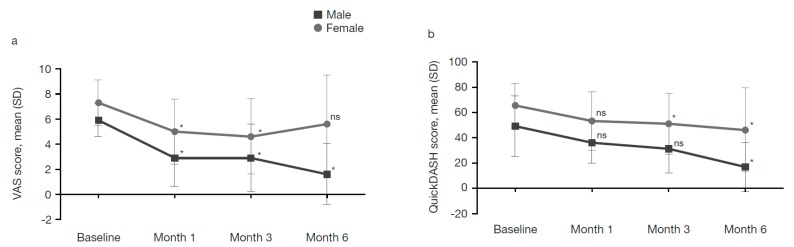
Pain intensity and functionality by visit and sex: (**a**) VAS score (pain); and (**b**) QuickDASH score (functionality). * Statistically significant difference between baseline and follow-up assessments at Months 1, 3 and 6; Tukey post-hoc analysis; *p* < 0.05. VAS range: 0 (best) to 10 (worst); QuickDASH scale range: 0 (best) to 100 (worst). SD, standard deviation; VAS, visual analogue scale. ns: not significant

The analysis of simple effects using a post-hoc Tukey test revealed no significant differences in VAS scores between male and female patients at baseline and at the first and second follow-up assessments (*p* > 0.05 for all). However, significant differences were observed between male and female patients at the third follow-up assessment (*p* < 0.05). The post-hoc Tukey test also showed that there were significant differences for VAS scores in the male group between baseline and first follow-up, baseline and second follow-up and baseline and third follow-up (*p* < 0.05 for all), while in the female group VAS scores were significantly different between baseline and first follow-up and baseline and second follow-up assessments only (*p* < 0.05 for both; Figure 3a). 

We also assessed differences in QuickDASH scores between male and female patients. A 2-way mixed ANOVA, where the between-subject factor was sex (2 levels) and the within-subject factor was the QuickDASH score at assessment visits (4 levels), showed main significant effects for sex and functionality of the upper limb (*p* < 0.05), indicating significant differences in both groups across the 4 time points. The Tukey post-hoc test revealed significant differences between baseline and first follow-up, baseline and second follow-up and baseline and third follow-up assessments (*p* < 0.05 for all) in both males and females. 

An analysis of the simple effects using the post-hoc Tukey test showed no significant differences in QuickDASH scores between male and female patients at baseline and at the first, second or third follow-up assessments (*p* > 0.05 for all). The post-hoc Tukey test showed that within the male group the QuickDASH scores were statistically significant only between baseline and third follow-up (*p* < 0.05). The post-hoc Tukey test also showed that within the female group the QuickDASH scores were significantly different between baseline and second follow-up (*p* < 0.05) and baseline and third follow-up (*p* < 0.05) assessments only (Figure 3b).

### 2.5. Safety

All patients treated in the extensor digitorum longus (21/24 patients [87.5%]) experienced weakness in the third finger following incobotulinumtoxinA treatment lasting between 45 and 90 days, consistent with previous reports. No adverse event was reported during the injection procedure and no adverse effects of treatment were reported at follow-up assessment visits. 

## 3. Discussion

Evidence in the available literature recommends the use of BoNT as a treatment for lateral epicondylitis refractory to other treatments and results are clearly superior to placebo [7,8,9,11], resulting in a grade A level recommendation based on 10 randomized clinical trials [13]. Variability resides in the duration of effects, with a clear recommendation to perform a careful muscle-by-muscle assessment [10].

In our study, the majority of patients (66.7%) were women. All patients were right handed, although in 20% of the cases the affected arm was not the dominant one, which could be associated with increased holding and loading carried out with that arm, leaving the more delicate handling to the dominant arm. The mean progression time between the initial symptoms until patients sought medical care was 20 months. All patients had received previous treatments. The most frequently affected muscles were the extensor digitorum longus (87.5%), the extensor carpi radialis brevis (83.3%), the extensor digiti minimi (41.7%) and the extensor carpi ulnaris (29.2%). Patients with >1 affected muscle received treatment in each of those muscles. This is in contrast to most other published studies, where patients only had a single muscle treated [10,11,14].

BoNT-A doses reported for treatment of lateral epicondylitis in other published studies are variable and may be reported as dose per muscle or total dose [7,14,15,16,17,18]; however, doses appear similar to those used in the present study. BoNT doses in the present study were administered in relation to muscle size and each patient received BoNT only in symptomatic muscles. Treated muscles did not display significant atrophy. 

The average duration of effect of the BoNT was around 3 months and weakness in the third finger was the main adverse effect, which is in agreement with previous literature [12]. Therefore, in patients for whom this side effect would impact on their ability to work, consideration should be taken before commencing treatment [14].

As a general outcome of the treatment, pain and upper limb function improved at the first follow-up assessment (1 month post-treatment) and this effect was maintained beyond the paralyzing effect of the toxin. Consistent with other studies [12], we observed a variation in functionality 3 months after the injection that appeared to correspond to the maximum effect and the maximum weakness of the extensor digitorum longus muscle. Weakness of this muscle may cause functional impairment to the patient, despite pain relief.

When data were stratified according to patients’ baseline VAS pain score (≥6 or <6 points), we found that improvements in pain and functionality were more pronounced in the group with VAS scores < 6, although only improvements in functionality reached significance. In this group, pain decreased by 46.7% and functional improvement was 50.3%, whereas in patients with VAS scores ≥ 6, improvements in both parameters were 36.8% and 37.5%, respectively. The higher residual pain intensity in patients with baseline VAS scores ≥ 6 may be explained by altered pain processing and central sensitization, associated with chronic musculoskeletal conditions including lateral epicondylitis [19]. However, the trend towards further improvement in functionality 6 months post-treatment in patients with VAS scores < 6, despite an increase in pain intensity, underscores pain as a symptom, rather than as the only factor contributing to functional impairment in lateral epicondylitis [20]. In the analysis of data stratified by sex, we found that men experienced a much more pronounced improvement in pain and functionality than women, consistent with known sex-related differences in pain perception and sensitivity [21]. However, these data could be statistically biased because of the difference in patient numbers resulting from stratification by sex (female, *n* = 16 vs. male, *n* = 8 patients) and VAS score (VAS ≥ 6, *n* = 18 vs. VAS < 6, *n* = 6).

No adverse events were registered during the study, apart from the expected weakness of the third finger. However, 3 patients (12.5%) received a repeat injection of incobotulinumtoxinA, since the treatment effect had been positive but of short duration. This may be due to the low dose used for the first injection. A further 5 (20.8%) patients were deemed to require surgical assessment as the effects of the BoNT treatment were insufficient to recover normal functionality.

Limitations of this experimental study include the small patient population and the disproportionate ratio of female to male patients in the study, which may limit the application of these findings to a broader population. Further studies in a larger patient population are warranted.

## 4. Conclusions

This study suggests that ultrasound-guided treatment with BoNT (incobotulinumtoxinA) in patients with lateral epicondylitis may be an effective treatment approach in the appropriate patient population. BoNT seems to be more effective in patients with baseline VAS score < 6 with epicondyle pain refractory to conservative therapy, thus being potential candidates for surgery. As such, these patients would be predicted to benefit most from BoNT treatment. Further studies are required to investigate the effectiveness of BoNT in other populations of patients with epicondylitis.

## 5. Materials and Methods 

### 5.1. Study Design

This was an experimental study of adult patients with epicondylitis, refractory to other treatments, who attended the Musculoskeletal Unit Rehabilitation Department, Hospital Universitario Virgen del Rocío between February 2014 and February 2015. The selected sample size was dependent on the number of patients attending the clinic.

As BoNT is not approved for the treatment of lateral epicondylitis, the Pharmacy Commission of the hospital was provided with the necessary Guideline for the Introduction of New Drugs in the Formulary form to obtain approval for this off-label use of BoNT. 

The study was approved by the Ethics Committee of the Hospital Universitario Virgen del Rocío and was conducted according to the Declaration of Helsinki (1964), approval code: 2014PI/055, approval date: 3 December 2014. Signed, informed consent was obtained from all patients.

### 5.2. Patients

Adult patients aged ≥ 18 years were eligible for inclusion in the study if they had lateral epicondylitis resistant or refractory to other treatments (including analgesics such as anaesthetic injections, anti-inflammatory drugs such as corticoids and physiotherapy), baseline pain intensity > 4 on a VAS ranging from 0 (best) to 10 (worst) [22]. and baseline functional impairment > 30 points on the QuickDASH scale ranging from 0 (best) to 100 (worst) [23].

Major exclusion criteria were any previous adverse reaction or allergy to treatment with BoNT, infectious disease with high fever and general discomfort, severe coagulation disorders, pregnancy or breastfeeding, medical history of concomitant diseases (such as renal or hepatic diseases) or neuromuscular diseases (such as myasthenia gravis or amyotrophic sclerosis) and previous treatment with aminoglycosides or other drugs that may interfere with neuromuscular junctions.

### 5.3. Study Treatment

Ultrasound-guided injections of incobotulinumtoxinA (reconstituted in 1 mL of normal saline) were administered at the predefined doses per muscle summarized in Table 2. Injections were given using insulin syringes in 1 site per muscle, with the exception of the extensor digitorum longus, in which the dose was split between 2 injection sites 4 cm apart. The target muscles for treatment were the extensor carpi ulnaris, the extensor digiti minimi, the extensor digitorum longus and the extensor carpi radialis brevis, selected on the basis of clinical symptoms, including the presence of pain during resisted movements. Patients with more than 1 affected muscle received treatment in each of these muscles.

The anatomical localization of muscles was determined by palpation from the epicondyle while the patient performed specific, requested movements, followed by ultrasound to ensure the correct placement of BoNT. Injections were performed through the long axis of the ultrasound probe. The affected muscles were preferentially injected in their motor end plates in order to control muscular hyperactivity as well as nociceptive neurotransmission. To avoid procedural variability, all injections were performed by the same experienced healthcare professional. Patients were assessed for treatment efficacy at clinical appointments at 1 month, 3 months and 6 months post-treatment.

Patients were permitted to take paracetamol (1 g every 8 h) for 48 h after incobotulinumtoxinA injection if they experienced pain or discomfort associated with the injection. Non-steroidal anti-inflammatory drugs and corticosteroids were not allowed during the study period. Patients were asked to avoid activities that required repetitive supination or strong compression and to interrupt any physiotherapeutic treatment.

### 5.4. Assessed Variables

The study captured information on the participating patients, including sociodemographic data and data related to disease progression, physical examination, pain (VAS; range 0 [best] to 10 [worst] [22]) and functionality (QuickDASH functional scale for upper limb; range 0 [best] to 100 [worst] [23]). Assessments were performed at the baseline treatment visit and at the assessment visits 1, 3 and 6 months post-treatment. Data on incobotulinumtoxinA doses administered, muscles treated and any reported adverse events were also collected.

### 5.5. Statistical Analysis

Data were analysed using descriptive statistics, employing absolute and relative frequencies for qualitative variables. Quantitative variables were presented as mean ± SD or median, interquartile range (P50 [P25–P75]), depending on whether or not the data were normally distributed (after applying the Shapiro-Wilk test [*n* < 50]). After evaluating the normality of data distribution, an ANOVA for repeated measurements, with estimations of the effect size and comparisons of the main effects, was performed to compare measurements at the different time points. As a secondary analysis, we stratified the population according to sex and baseline VAS score (≥6 or <6). Quantitative comparisons between groups were performed using 2-way mixed model ANOVAs and Tukey post-hoc tests were developed. The differences were considered significant at *p* < 0.05. Data were analysed using IBM SPSS Statistics 22 (IBM Corp, Armonk, NY, USA).

## Figures and Tables

**Table 1:** Patient baseline demographics and characteristics.

Patients	*n* = 24
Sex	*n* (%)
Male	8 (33.3)
Female	16 (66.7)
	Age years
Mean (SD)	46.8 (9)
Median (interquartile range)	48.5 (43.0 to 52.8)
Patients with right dominance, n (%)	24 (100)
Injured side	*n* (%)
Left	5 (20.8)
Right	19 (79.2)
	Symptom progression time, months
Mean (SD)	20.0 (19.8)
Median (interquartile range)	12 (9.3 to 24.0)
	Pain, baseline VAS score
Mean (SD)	6.85 (1.8)
Median (interquartile range)	7 (5.3 to 8.4)
	Upper limb functionality, baseline QuickDASH score
Mean (SD)	60.12 (20.9)
Median (interquartile range)	60 (45.0 to 79.5)
Previous ineffective treatments	*n* (%)
Analgesics/NSAIDs	24 (100)
Physiotherapy	17 (70.8)
Electrotherapy	13 (54.2)
Peritendinous injections	20 (83.3)
Positive resisted manoeuvres at baseline	*n* (%)
Extensor carpi ulnaris	7 (29.2)
Extensor digiti minimi	10 (41.7)
Extensor digitorum longus	21 (87.5)
Extensor carpi radialis brevis	20 (83.3)

VAS range: 0 (best) to 10 (worst); QuickDASH scale range: 0 (best) to 100 (worst). NSAID, non-steroidal anti-inflammatory drug; SD, standard deviation; VAS, visual analogue scale.

**Table 2:** IncobotulinumtoxinA doses per muscle.

Muscle Treated	IncobotulinumtoxinA Dose, U	Number of Patients Treated, *n* (%)
Extensor carpi ulnaris	20	7 (29.2)
Extensor digiti minimi	10	9 (37.5)
Extensor digitorum longus	30	21 (87.5)
Extensor carpi radialis brevis	20	20 (83.3)

Doses were predefined for each muscle based on muscle size. Patients could receive treatment in > 1 muscle if clinically required.
